# Deregulation of complement components *C4A* and *CSMD1* peripheral expression in first-episode psychosis and links to cognitive ability

**DOI:** 10.1007/s00406-022-01409-5

**Published:** 2022-05-09

**Authors:** Alex Hatzimanolis, Stefania Foteli, Pentagiotissa Stefanatou, Angeliki-Aikaterini Ntigrintaki, Irene Ralli, Konstantinos Kollias, Chrysoula Nikolaou, Maria Gazouli, Nikos C. Stefanis

**Affiliations:** 1 Department of Psychiatry, School of Medicine, National and Kapodistrian University of Athens, Eginition Hospital, 72 Vas. Sophias Ave., 115 28 Athens, Greece; 2 Neurobiological Research Institute, Theodor-Theohari Cozzika Foundation, 69-71 Souidias St., 115 21 Athens, Greece; 3 Department of Biopathology and Immunology, School of Medicine, National and Kapodistrian University of Athens, Eginition Hospital, 72 Vas. Sophias Ave., 115 28 Athens, Greece; 4 Laboratory of Biology, Department of Basic Medical Sciences, School of Medicine, National and Kapodistrian University of Athens, 176 Michalakopoulou Ave., 115 27 Athens, Greece; 5 School of Science and Technology, Hellenic Open University, 18 Aristotelous St., 263 35 Patras, Greece

**Keywords:** *C4A*, *CSMD1*, Gene expression, First-episode psychosis, Schizophrenia, Cognition

## Abstract

Up-regulation of the complement component 4A (C4A) in the brain has been associated with excessive synaptic pruning and increased schizophrenia (SZ) susceptibility. Over-expression of *C4A* has been observed in SZ postmortem brain tissue, and the gene encoding for a protein inhibitor of C4A activity, CUB and Sushi multiple domains 1 (*CSMD1*) gene, has been implicated in SZ risk and cognitive ability. Herein, we examined *C4A* and *CSMD1* mRNA expression in peripheral blood from antipsychotic-naive individuals with first-episode psychosis (FEP; *n* = 73) and mentally healthy volunteers (*n* = 48). Imputed *C4* locus structural alleles and C4A serum protein levels were investigated. Associations with symptom severity and cognitive domains performance were explored. A significant decrease in *CSMD1* expression levels was noted among FEP patients compared to healthy volunteers, further indicating a positive correlation between *C4A* and *CSMD1* mRNA levels in healthy volunteers but not in FEP cases. In addition, *C4* copy number variants previously associated with SZ risk correlated with higher *C4A* mRNA levels in FEP cases, which confirms the regulatory effect of *C4* structural variants on gene expression. Evidence also emerged for markedly elevated C4A serum concentrations in FEP cases. Within the FEP patient group, higher *C4A* mRNA levels correlated with more severe general psychopathology symptoms and lower *CSMD1* mRNA levels predicted worse working memory performance. Overall, these findings suggest C4A complement pathway perturbations in individuals with FEP and corroborate the involvement of *CSMD1* in prefrontal-mediated cognitive functioning.

**Supplementary Information:**

The online version contains supplementary material available at 10.1007/s00406-022-01409-5.

## Introduction

A large number of investigations have reported biochemical alterations of the immune system in patients with schizophrenia (SZ), providing support of the immune/inflammatory hypothesis for SZ as a putative pathophysiological mechanism increasing the vulnerability to the illness [1–3]. Similarly, dysregulated immune/inflammatory responses and differential expression of immune-related genes have been observed in individuals with first-episode of psychosis (FEP) [4–7], indicating that immune aberrations may exist even at the early stages of psychosis, further corroborating the view that underlying immunological deficiencies could play a role in the development or progression of psychotic disorders [8, 9]. In line with the above notion, genetic evidence has also emerged highlighting the involvement of genes coding for immune system components in SZ pathology [10, 11]. The involvement of complement system alterations in SZ etiopathogenesis, likely through the increased activity of the classical complement pathway, has long been considered an indication of disturbed innate immunity in SZ which may negatively affect neurodevelopmental processes [12]. On another front, the exacerbation of immune/inflammatory reactions during the acute phase of psychotic illness could be viewed as a compensatory or protective physiological mechanism that may alleviate psychotic symptoms and cognitive impairment [13]. Environmental influences and psychosocial distress may also induce secondary physiological processes including an increase of inflammatory responses [14, 15].

From a genomic perspective, fine-mapping of associations derived from large-scale genome-wide association studies (GWAS) in SZ, suggested that the complement component C4 constitutes a potential immune mediator which increases SZ susceptibility [11]. Specifically, structurally distinct alleles at the C4 gene locus, which encodes the complement component C4, have been genetically linked to SZ risk and associated with elevated *C4A* isotype gene expression in postmortem brain tissue from SZ patients. Over-expression of *C4*/C4A mRNA transcripts has been observed in multiple brain regions of SZ patients compared to healthy individuals [16, 17]. It is noted that C4 protein product is a member of the classical complement pathway that is activated during the innate immune response and forms a proteolytic protein cascade that clears cellular debris, enhances inflammation, and it is involved in the engulfment or elimination of pathogens [18]. Moreover, evidence from animal studies indicates that higher *C4A* isotype expression is implicated in excessive synaptic pruning in the brain, likely contributing to behavioral disturbances and cognitive deficits [19]. In accordance with the aforementioned findings, higher genetically predicted *C4* gene expression is associated with poor memory performance in both SZ patients and healthy individuals [20]. With respect to differences in psychotic symptoms, increased *C4A* mRNA levels in peripheral blood cells have been shown to correlate with greater severity of psychopathology in SZ patients, specifically delusional symptoms [21]. Additionally, up-regulation of C4A protein levels has been found in plasma as well as in cerebrospinal fluid from patients with SZ [22–25]. Preliminary findings also suggest that higher total C4 plasma levels may predict unfavorable treatment response among FEP patients followed-up for twelve months [26].

It is of interest that among the strongest associated loci with SZ in recent GWAS is the CUB and Sushi Multiple Domains 1 (*CSMD1*) genetic locus, which encodes a protein inhibitor of C4 activity in neural tissues [27–29]. Further, reduced *CSMD1* mRNA expression has been observed in peripheral blood of SZ patients [30] and common genetic variation within *CSMD1* has been associated with psychosis proneness in the general population [31], as well as memory functioning in SZ and healthy individuals [32, 33]. Prompted by earlier findings implicating *C4A* and *CSMD1* peripheral aberrations in SZ, the aim of the present study was to examine *C4A* and *CSMD1* mRNA expression levels, as well as serum C4A protein levels, in peripheral blood from well-characterized antipsychotic-naïve FEP cases and mentally healthy individuals. In addition, genetically predicted *C4* expression was estimated and we evaluated whether gene expression correlates with symptom severity at the early course of the illness and cognitive performance.

## Materials and methods

### Participants

In the current study, we included 73 unrelated cases (mean age 25.0 ± 7.2 years; 69% males) with non-affective first-episode psychosis (FEP) recruited as part of the collaborative Athens FEP Research Study [34]. Clinical diagnoses were established based on the International Classification of Diseases 10th Revision (ICD-10) diagnostic criteria (WHO, 1992) [35]. Consensus diagnoses for all cases were obtained from trained psychiatrists on the basis of detailed clinical records and individuals fulfilling diagnostic criteria for Schizophrenia-spectrum disorders (ICD-10 codes: F20-F29) were examined. All cases at the time of admission were antipsychotic-naïve and blood collection sampling was performed for basic biochemical examination and subsequent genomic analysis. Symptom severity at admission was assessed using the Positive and Negative Syndrome Scale (PANSS) [36]. Of the 73 FEP cases included in the study, 58 (79.5%) were hospitalized. Detailed demographic and clinical information is presented in Table S1. A total of 48 unrelated healthy volunteers (mean age 26.5 ± 4.8 years; 60.4% males) with no history of psychiatric disorder donated blood samples for annual routine biochemical examination and served as the control group. Each volunteer underwent a brief medical interview from trained physicians to assess the presence of major mental illness and other neurological or immunological disorders. Written informed consent was obtained from every individual after a detailed description of the research objectives and the study protocol was approved by the ethics committee and the Institutional Review Board at Eginition University Hospital (Athens, Greece).

### Cognitive assessment

General cognitive ability was estimated using the Greek version of the Wechsler Adult Intelligence Scale (WAIS-Fourth edition) [37, 38], a comprehensive neurocognitive test comprising ten basic subtests grouped into four indexes representing distinct cognitive domains: verbal comprehension, perceptual reasoning, working memory and processing speed. Subtest raw scores were converted into age-corrected scaled scores using available Greek norms to determine the individual index scores and the full-scale IQ score. The assessment of neurocognitive functioning was performed within 3–4 weeks following admission to the research protocol by trained clinical neuropsychologists at Eginition University Hospital. In the current study, we enrolled 59 FEP cases with available neuropsychological data for further analysis.

### RNA extraction and gene expression analysis

Total RNA was extracted from peripheral blood mononuclear cells (PBMCs) using the NucleoSpin RNA Blood kit (Macherey–Nagel, Düren, Germany), according to the protocol provided by the manufacturer. The purity and integrity of total RNA were evaluated using UV measurements and denaturing agarose gel electrophoresis, respectively. Reverse transcription (RT) reactions were performed using the PrimeScript First-Strand cDNA Synthesis Kit (TAKARA Bio, Japan) at 37 °C for 30 min followed by 85 °C for 5 s. The mRNA expression levels of *C4A* and *CSMD1* were measured using semi-quantitative real-time polymerase chain reaction (RT-qPCR) on an ABI Prism 7000 instrument (Applied Biosystems, Foster City, CA, USA). Every cDNA sample was mixed with specific sets of primers and the qPCR master mix (KAPA SYBR FAST Universal Kit, Sigma-Aldrich, Germany) for 2 min at 50 °C and 2 min at 95 °C, followed by 40 cycles consisting of 15 s at 95 °C and 60 s at 60 °C. Finally, a standard dissociation protocol was used to ensure that each amplicon was a single product. All reactions were held in triplicate to ensure reproducible results. To evaluate differences in gene expression between groups, the fold change was calculated for each gene applying the comparative Ct (2^−∆∆Ct^) method. Relative mRNA expression levels were estimated by calculating delta Ct (Cycle threshold) values using the Ct values of the *GAPDH* housekeeping gene for normalization. Under-expressed genes are shown as the negative inverse of fold change and over-expressed as the fold change. Primers sequences were as follows: *C4A* forward, 5′-GGCTCACAGCCTTTGTGTTG-3′; *C4A* reverse, 5′-CCCTGCATGCTCCTGTCTAA-3′; *CSMD1* forward, 5’-GTCTGGGCTCGTGGATATGT-3’; *CSMD1* reverse, 5’-CAGGTCTCGGAAGGACAGAG-3’ and *GAPDH* forward, 5′-CGAGATCCCTCCAAAATCAA-3′; *GAPDH* reverse, 5′-TTCACACCCATGACGAACAT-3′.

### Genotyping and C4 structural allele imputation

Genome-wide genotyping of unrelated FEP cases was performed using the Infinium Omni2.5 BeadChip array (Illumina Inc., San Diego, USA) at the Johns Hopkins Center for Inherited Disease Research (CIDR). Standard quality control (QC) processing steps of genotype data were performed using PLINK v1.90 [39]. High-quality genotyped single-nucleotide polymorphisms (SNPs) with call rate > 95%, minor allele frequency > 1%, Hardy–Weinberg equilibrium deviation p > 10^–6^ were retained for further analyses. Samples with low genotype rates (< 95%) were also excluded. To infer *C4* structural alleles, 12,052 SNPs spanning the MHC region were extracted and utilized for imputation of *C4* copy number structural variants following the procedures previously described by Sekar et al*.* (2016), using the MHC haplotypes HapMap3 CEU reference panel as recommended by the aforementioned group (http://mccarrolllab.org/resources/resources-for-c4/). Four common *C4* haplotype groups were derived (BS, AL-BS, AL-BL, AL-AL) based on the combination of the *C4* structural elements (C4A, C4B, C4L, C4S) that each individual carries and genetically predicted *C4A* expression levels were estimated in accordance with prior studies [11, 40]. In our FEP sample, the *C4A* predicted expression values ranged between 0 and 1.61 (mean: 1.26; standard deviation: 0.22).

### Quantification of C4A serum levels

Human complement C4A protein levels in serum samples were determined using an enzyme-linked immunosorbent (ELISA) assay kit (AssayGenie, Dublin, Ireland) following the manufacturer’s instructions. Peripheral blood samples were obtained from every participant at admission (between 8.00 and 11.00 am) and centrifuged within 30 min after blood draw to collect serum samples for routine biochemical analysis. Serum aliquots were kept frozen at − 70 °C until further analysis. ELISA measurements were performed in a subset of FEP cases (*n* = 62) with available serum samples and triplicates were tested for each sample to calculate C4A mean concentration values for downstream statistical analyses.

### Statistical analyses

Demographic characteristics were compared between groups by applying either Pearson’s chi-square (categorical variables) or Mann–Whitney *U* tests (continuous variables), as appropriate. To evaluate gene expression differences between FEP cases and healthy volunteers, the comparative Ct method (2^−∆∆Ct^) was utilized and fold-change differences were calculated. Differences in relative mRNA expression levels (log-transformed ΔCt values) between groups and C4A serum levels were examined using one-sided Mann–Whitney *U* tests, as prior evidence has shown altered expression levels of *C4A* and *CSMD1* in SZ [11, 16, 30]. Within-group correlations between *C4A* and *CSMD1* relative gene expression were tested by applying Spearman’s rank correlation coefficients (rho). Linear regression models adjusted for age and gender were applied to test for associations between relative gene expression levels and PANSS subscale scores or WAIS-IV cognitive domains scores. Correlations between genetically predicted *C4* copy number structural alleles (*C4* haplotype groups) and mRNA transcript levels were estimated by linear regression analysis as previously reported (40). The significance threshold was set at *p* < 0.05. All analyses were performed using R version 4.1.2 package.

## Results

### Demographic and clinical characteristics

In the present study, we included a total of 73 FEP cases and 48 mentally healthy volunteers. Detailed demographic and clinical information for the FEP group of cases is presented in Supplementary Table S1. No significant differences were observed with regard to gender (*χ*^2^ = 0.515; *p* = 0.473) or age (Mann–Whitney test *p* = 0.205) between FEP and healthy groups. All FEP cases were antipsychotic-naïve at the time of inclusion to the study and 80% were hospitalized. According to the ICD-10 diagnostic criteria, 88% of cases received a Schizophrenia (SZ) diagnosis (F20 ICD-10 code). The remaining cases (12%) were classified as psychosis-spectrum disorders (F23 or F28 ICD-10 codes; Supplementary Table S1).

### C4A and CSMD1 mRNA expression levels in peripheral blood

**Fig. 1:**
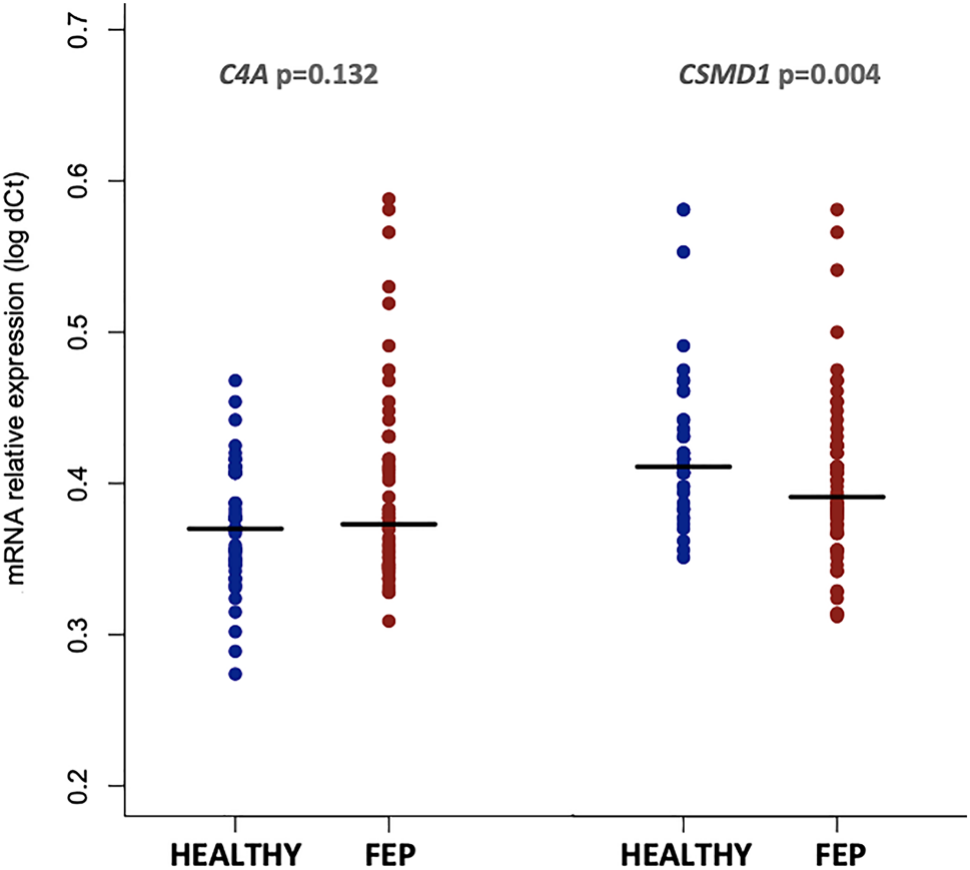
Relative mRNA expression levels for **A** *C4A* and **B** *CSMD1* between healthy volunteers and FEP cases. Horizontal black lines indicate the median value for each group. *P* values were derived by a one-sided non-parametric Mann–Whitney test

The relative expression of *C4A* and *CSMD1* mRNA levels in PBMCs was compared between FEP cases and healthy volunteers. As shown in Fig. 1, we observed that *C4A* was marginally over-expressed in FEP cases; however, this difference did not reach statistical significance (one-sided Mann–Whitney test *p* = 0.132). *CSMD1* gene expression was found significantly reduced among FEP cases compared to healthy volunteers (1.4-fold change; one-sided Mann–Whitney test *p* = 0.004). Female FEP cases showed a trend toward higher *C4A* expression levels compared to male cases (Mann–Whitney test *p* = 0.067), whereas the above association was not present in healthy participants (Mann–Whitney test *p* = 0.749). Sex differences were not observed for *CSMD1* expression levels in both FEP and healthy groups. Furthermore, all the above associations remained unchanged after adjustment for ICD-10-based diagnostic classifications. Within-group analyses revealed a significant positive correlation between *C4A* and *CSMD1* mRNA expression levels in healthy volunteers (rho = 0.40, *p* = 0.005), yet this relationship was not detected in FEP cases (rho = 0.16, *p* = 0.197).

### C4 structural genetic variation correlates with C4A mRNA levels

**Fig. 2:**
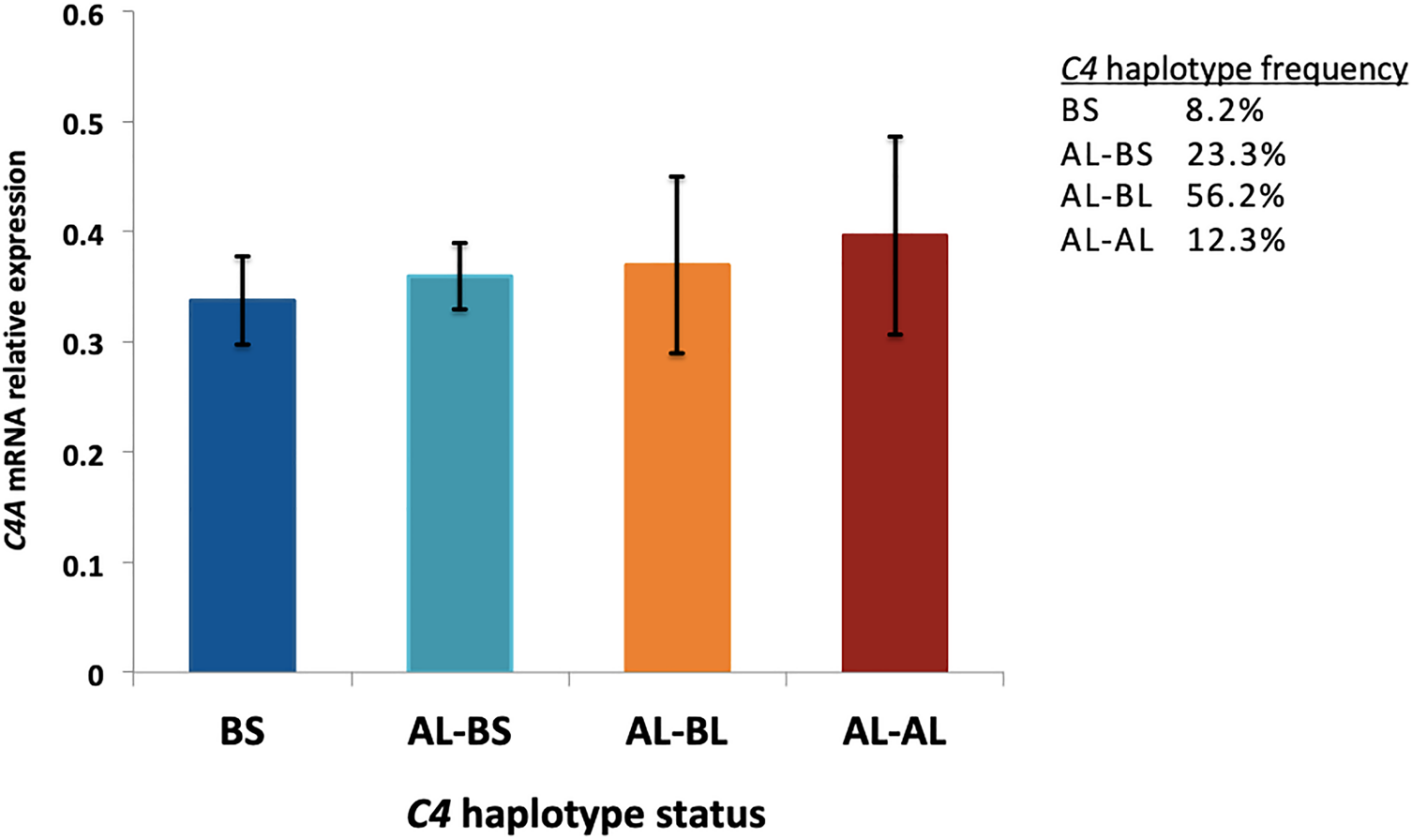
Correlation between *C4* locus structural alleles (*C4* haplotype groups) and mRNA expression levels in peripheral blood from FEP cases. Mean expression level (± standard error) is shown for each haplotype group

We further examined the correlation between *C4A/C4B* locus structural alleles (haplotype groups: BS, AL-BS, AL-BL, AL-AL) and measured mRNA levels in peripheral PBMCs, in an effort to validate previous findings in the human brain [11], demonstrating an association between genetically predicted *C4A/C4B* haplotype status and gene expression. Our results indicated that among FEP cases (*n* = 71), carriers of the SZ risk AL-AL haplotype showed higher *C4A* mRNA levels (*β* = 0.26; *p* = 0.02) (Fig. 2), whereas no association was found with *CSMD1* mRNA levels (*β* = − 0.03; *p* = 0.817), as expected. In agreement with recent evidence from an independent FEP study [22], we observed that the AL-BL haplotype group was the most common among FEP cases (56%), compared to a much lower frequency reported within healthy individuals (41%) in the original study by Sekar et al*.* (2016). Likewise, the SZ low risk BS haplogroup (7% frequency in the Sekar et al. study) reached a comparable frequency in this FEP sample (8.2%).

### Elevated C4A serum levels in FEP cases

**Fig. 3:**
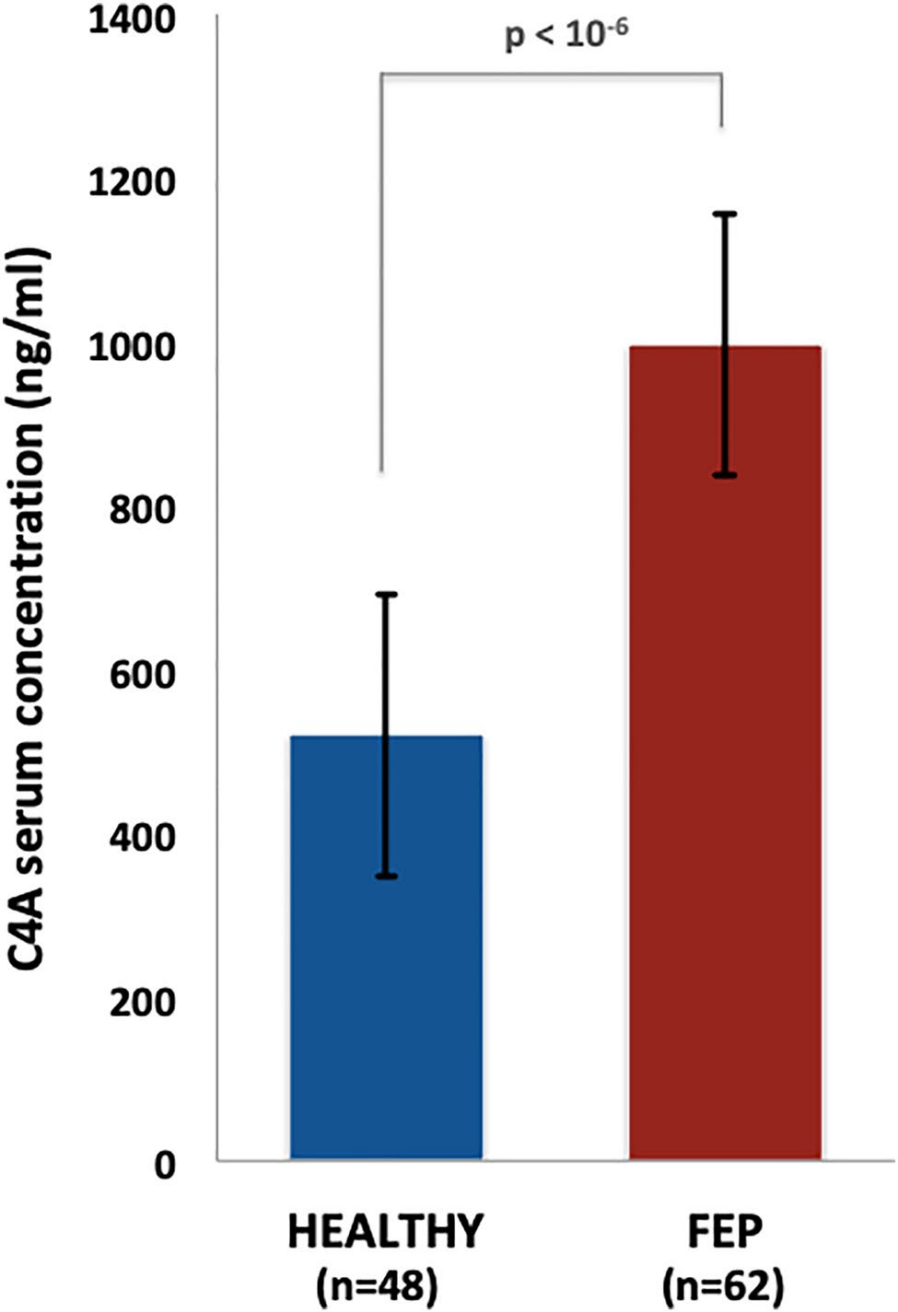
Serum C4A protein levels measurements. Error bars represent standard deviation and statistical significance was derived by a non-parametric Mann–Whitney test

The C4A protein concentrations were found significantly elevated in FEP cases compared to healthy volunteers (mean FEP: 999.5 ± 160.9 ng/ml; mean healthy: 519.4 ± 172.6 ng/ml, Mann–Whitney test *p* < 10^–6^; Fig. 3). C4A serum levels did not differ significantly by gender (*χ*^2^ = 115.8; *p* = 0.359) or age (Mann–Whitney test *p* = 0.906). We also noted that *C4A* blood mRNA levels did not correlate significantly with C4A serum levels in our sample (*β* = 0.12; *p* = 0.421). Similarly, genetically predicted *C4A* expression (*C4* copy number variation) was not associated with C4A protein levels (*β* = 0.05; *p* = 0.747).

### Associations with symptom severity and cognitive performance

**Fig. 4:**
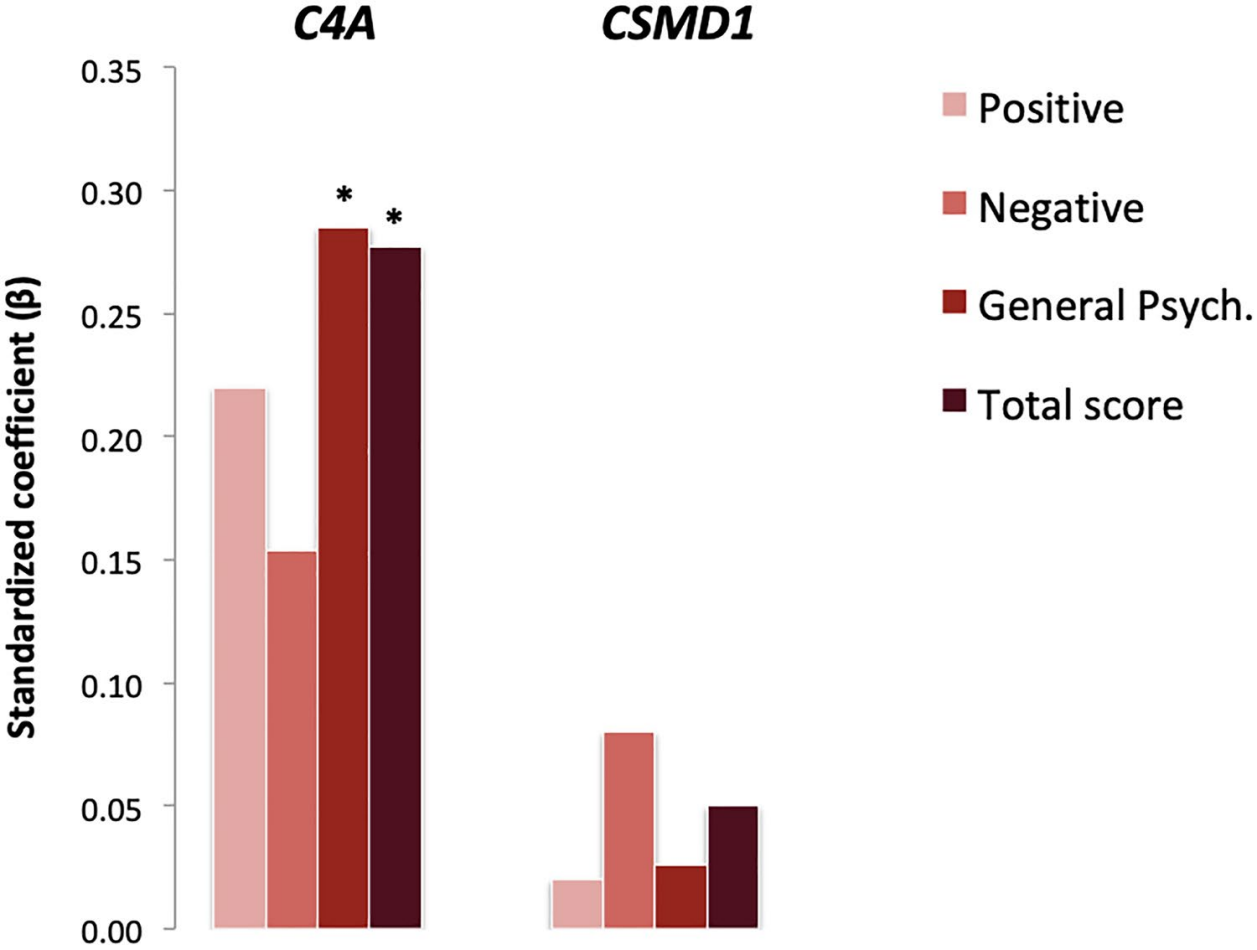
Association between *C4A*, *CSMD1* peripheral gene expression levels and PANSS subscale severity scores at admission among patients with FEP (*two-sided *p* < 0.05)

**Fig. 5:**
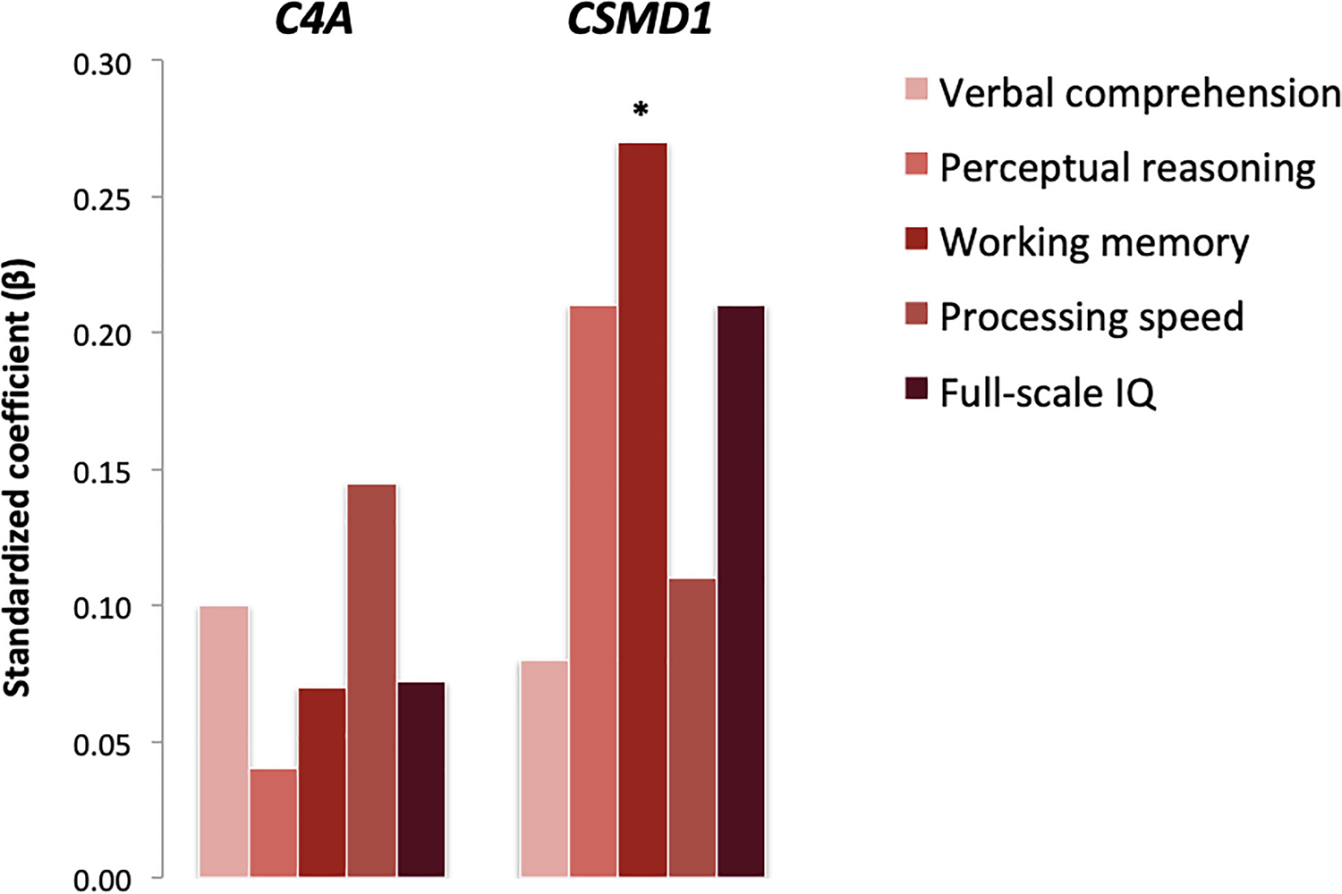
Association between *C4A*, *CSMD1* peripheral gene expression levels and WAIS-IV assessed neuropsychological indexes in patients with FEP (*two-sided *p* < 0.05)

Within-group analyses were applied to evaluate the potential impact of *C4A* and *CSMD1* gene expression on PANSS subscales scores at admission as well as cognitive performance in FEP cases. As shown in Fig. 4, significant positive correlations were observed between *C4A* mRNA expression levels and PANSS general psychopathology (*β* = 0.29; *p* = 0.016) and total (*β* = 0.28; *p* = 0.019) symptom scores. With regard to cognitive performance, increased *CSMD1* mRNA levels were associated with better performance on working memory (*β* = 0.27; *p* = 0.037). *C4A* mRNA and C4A serum protein levels did not correlate significantly with either PANSS baseline subscales scores or cognitive indices (Fig. 5; Supplementary Table S2).

## Discussion

The results of the present study add to a growing body of evidence implicating aberrant immune/inflammatory responses in patients with SZ and those experiencing FEP [1, 4–6, 26, 41]. We provide evidence for common transcriptional regulation of *C4A* and *CSMD1* among healthy individuals, suggesting co-expression of the two genes and related biological functions in the C4A-dependent complement pathway. Importantly, our results indicate C4A cascade alterations in un-medicated cases with FEP, suggesting an abnormal innate immune reaction during the early course of psychosis [5, 26]. It is stressed, though, that there is a difficulty to precisely identify the exact etio-pathological mechanisms which induce C4 complement activation and related immunological dysregulation in FEP. The relationship between environmental stress or adverse life events and the enhancement of immune/inflammatory responses has been documented in psychotic disorders [14, 42, 43], and plausibly explains the immune exacerbations following an acute episode of psychosis [13]. Moreover, up-regulation of the complement system has been observed in rodents following exposure to stressful conditions [44]. The observed gene expression differences in FEP patients are in accordance with previous studies indicating higher *C4*/*C4A* expression levels in postmortem brain tissue [11, 16, 17] and significantly lower *CSMD1* expression levels in peripheral blood from SZ patients [30]. A trend for increased *C4A* expression levels in PBMCs, although not statistically significant, has been observed in an earlier study which examined *C4A* blood mRNA levels in patients with SZ and psychotic bipolar disorder under medication with antipsychotics [21]. We argue that antipsychotic treatment could potentially impact gene expression levels; therefore, future investigations with larger sample sizes and well-characterized un-medicated patients are needed to delineate whether increased *C4A* expression stems from illness specific etio-pathological mechanisms or unidentified non-specific drug-induced phenomena.

This is to the best of our knowledge the first study reporting *C4A* isotype mRNA expression levels in peripheral blood in relation to genetically predicted *C4A* gene expression. In particular, we validate the positive correlation previously seen in brain postmortem tissue between SZ risk increasing *C4A* structural variation (i.e., AL-AL haplotype) and experimentally determined mRNA levels in immune cells [11]. The above observation supports the regulatory effect of distinct *C4A* copy number variants on gene transcription and has significant implications for future genetic studies aiming to estimate *C4A* expression profiles and investigate the contribution of deregulated *C4/C4A* expression on psychotic disorders. It is of interest that prior evidence has shown that genetically predicted *C4A* expression associates with cognitive impairment in SZ patients and differences in brain imaging measures, such as cortical activation and thickness in healthy individuals [20, 40]. In this work, there was no indication for a correlation between either predicted *C4* structural alleles or measured mRNA levels and cognitive performance among FEP cases. Notably, the most frequent *C4* structural variant, that is AL-BL haplogroup, was associated with higher SZ risk in the original study by Sekar et al*.* (2016) and was found to predispose to microbial infections in SZ patients [45], which naturally could activate innate immune responses and complement system up-regulation.

In addition, we provide evidence that FEP cases are characterized by highly elevated serum C4A protein levels, compared to mentally healthy volunteers. This observation confirms the results of an earlier study reporting increased C4A levels in SZ patients measured by highly sensitive mass spectrometry methodology [23]. It is mentioned, however, that most studies so far have estimated peripheral total C4 protein levels in cases diagnosed with FEP or SZ reporting no significant differences in serum [24, 25, 46], but elevated levels in cerebrospinal fluid [24]. Our findings outline that a specific dysregulation of C4A isotype levels may exist in psychosis, at least in the very early stages of the illness, which could not be detected using methods that estimate total amount of complement C4 (i.e., C4A and C4B levels) [6]. It remains to be determined in future studies whether the observed increase in C4A protein levels is attributed to underlying biochemical alterations with considerable biological impact on the disposition to psychosis. To this perspective, findings from FEP cases followed-up for an entire year showed that higher baseline C4 serum levels may represent an early indicator of poor treatment response [26], suggesting that peripheral C4 concentration could potentially inform clinicians for optimal treatment intervention in individuals with FEP. Our analyses did not reveal significant correlation between C4A serum protein and mRNA levels in this population, which is possibly attributed to complicated cellular mechanisms involved in gene transcription regulation, mRNA processing and protein synthesis rates [47, 48]. Moreover, we acknowledge as an additional limiting factor the slightly smaller number of FEP cases included in the C4A protein assays that might have reduced the statistical power of the analysis. Likewise, *C4* haplogroup analysis among FEP cases did not reveal an association between *C4A* copy number status and C4A protein levels in serum, as opposed to an earlier study which examined a limited number of plasma samples from medicated SZ patients [22].

Prompted by previous reports supporting a relationship between *C4A* and *CSMD1* gene expression deviations and symptom dimensions as well as cognitive function [20, 21, 30, 49], we attempted to assess the relationships with symptom severity and cognitive performance in cases with FEP. In our sample, nominally significant associations were noted between *C4A* peripheral mRNA levels and PANSS general psychopathology as well as total symptoms severity, which corroborate prior evidence indicating a relationship between higher *C4A* expression levels in PBMCs and more severe psychotic symptomatology [21], albeit it should be stressed that our limited sample size could not permit definite conclusions. Further, recent findings from a FEP cohort did not observe correlations between genetically predicted *C4* expression by estimating *C4* haplotype status and symptom severity [22], implying that additional evidence is essential to elucidate whether altered C4/C4A expression could prove as a meaningful clinical biomarker [50].

Importantly, our findings indicate that lower peripheral *CSMD1* expression levels correlate with poor performance on prefrontal-mediated cognitive domains, in particular perceptual reasoning. Common genetic variation within *CSMD1* has been credibly associated with SZ in recent GWAS meta-analyses [51], and implicated in human cognitive impairment as well as reduced brain functional activation [33, 52]. We postulate that lower *CSMD1* expression negatively impacts on cognitive functioning in FEP cases by compromising C4-dependent complement pathway, which has been involved in synaptic pruning processes and neurodevelopmental mechanisms [11, 53]. It is noteworthy to mention that functional studies have reported that CSMD1 constitutes a complement cascade regulatory factor, acting as an inhibitor of C4-dependent cellular processes in neural tissues [28, 29]. The biological relationship between C4A and CSMD1 components and their role in shared biochemical processes of the complement system is strengthened by studies examining psychosis-related behavioral and cognitive phenotypes in mice. Specifically, over-expression of C4/C4A components might contribute to the enhancement of anxiety-like behaviors, social impairment and working memory deficits [19, 54], whereas depletion of the *CSMD1* genetic locus induces emotional and cognitive defects [55]. Therefore, it becomes evident that C4A and CSMD1 complement factors likely operate in common cellular pathways with opposing functions [29]. To this perspective, the observed inverse direction of gene expression patterns for *C4A* and *CSMD1* in FEP cases denotes a transcriptional dysregulation of the two complement-related genes at psychosis onset and/or at a drug-naïve state.

In conclusion, this study suggests a hyperactivity of the C4/C4A complement pathway in un-medicated individuals diagnosed with FEP, predominantly SZ [12, 56]. Furthermore, the results add further support to an altered immune/inflammatory response at least in a subset of individuals with FEP, which might contribute to the development of psychotic symptoms [5, 6, 9]. The exact biological importance of complement pathway dysregulation in the occurrence of psychosis is still not well understood [50], although recent evidence has implicated complement system alterations to early-life brain synaptic abnormalities and neurodevelopmental deficits, perhaps related to increased SZ predisposition [53, 56, 57]. Additional research efforts aiming to characterize the immune/inflammatory profile early in the course of psychotic illness may shed more light on the influence of complement factors in the pathogenesis of SZ and related psychosis-spectrum disorders [56].

## Supplementary Information

Below is the link to the electronic supplementary material.

Supplementary file1 (DOCX 41 kb)
