# Supplementary material for: Deregulation of complement components C4A and CSMD1 peripheral expression in first-episode psychosis and links to cognitive ability
Source: Eur Arch Psychiatry Clin Neurosci. 2022 May 9;272(7):1219–28. doi: 10.1007/s00406-022-01409-5 (PMC9508018; doi:10.1007/s00406-022-01409-5)
Supplement: Supplementary file 1 — Supplementary file1 (DOCX 41 kb) [file 406_2022_1409_MOESM1_ESM.docx]

**SUPPLEMENTARY MATERIAL**

**Supplementary Table S1.** Demographic and clinical charecteristics of first-episode psychosis (FEP; N=73) cases included in the study

| **Gender**  **(Males/Females)** | 50 / 23 |
| --- | --- |
| **Age**  **(Mean±SD)** | 25.0 (7.2) |
| **Schizophrenia**  **(ICD-10 F20, %)** | 88.0 |
| **Brief psychotic disorder**  **(ICD-10 F23, %)** | 6.9 |
| **Other psychotic disorder**  **(ICD-10 F28, %)** | 5.1 |
| **Antipsychotic-naïve**  **at admission (%)** | 100 |
| **Inpatients (%)** | 80.0 |
| **PANSS subscales scores at admission (Mean±SD)** | |
| **Positive** | 28.5 (6.4) |
| **Negative** | 22.2 (9.5) |
| **General**  **Psychopathology** | 50.0 (15.1) |
| **Total score** | 100.7 (26.0) |

**Supplementary Table S2:** Association between C4A serum protein levels, symptom severity at admission and cognitive domains

|  | **C4A serum protein levels** | |
| --- | --- | --- |
| **PANSS subscale** | **β coefficient** | **p-value** |
| Positive | 0.108 | 0.418 |
| Negative | 0.014 | 0.908 |
| General psychopathology | 0.088 | 0.503 |
| Total score | 0.084 | 0.511 |
| **WAIS-IV subscale** |  |  |
| Verbal comprehension | 0.015 | 0.914 |
| Perceptual reasoning | -0.183 | 0.194 |
| Working memory | -0.152 | 0.256 |
| Processing speed | 0.028 | 0.827 |
| Full-scale IQ | -0.126 | 0.341 |
